# Wearable Sensor–Derived Gait Parameters Across Self-Reported Physical Activity Levels in Individuals With Knee Osteoarthritis and Healthy Controls: Pilot Cross-Sectional Validation Study

**DOI:** 10.2196/80728

**Published:** 2026-06-03

**Authors:** Charles Amoatey Odonkor, Siri Bohacek, Salman Hirani, Wei Zhang, Amir Muaremi, Heike Leutheuser, Matthew Smuck

**Affiliations:** 1 Division of Physiatry, Department of Orthopedics and Rehabilitation, Yale University, 47 College Street, New Haven, CT, 06520, United States, 1 203-737-5484, 1 203-785-7132; 2 Department of Anesthesia and Perioperative Medicine, Oregon Health & Science University, 3303 S Bond Ave, Portland, OR, 97239, United States; 3 Laboratory of Movement Analysis and Measurement, Ecole Polytechnique Fédérale de Lausanne, Lausanne, Switzerland; 4 Translation Medicine Department, Novartis Institutes for BioMedical Research, Basel, Switzerland; 5 Chair of Machine Learning in Medicine, Department of Computer Science, University of Bayreuth, Bayreuth, Germany

**Keywords:** knee osteoarthritis, wearable sensors, gait analysis, physical activity, feasibility study, inertial measurement units, mobility assessment

## Abstract

**Background:**

Wearable inertial measurement units (IMUs) offer scalable, objective gait assessment, but detailed stride-level validation against motion capture and their ability to reflect physical activity (PA)–related gait differences in knee osteoarthritis (KOA) remain incompletely characterized.

**Objective:**

This study aimed to evaluate the technical validity of foot-mounted IMU–derived gait parameters compared with optical motion capture in individuals with KOA and healthy controls. As a secondary exploratory aim, we assessed whether IMU-derived gait parameters differed across self-reported PA levels.

**Methods:**

In this pilot cross-sectional validation study, 20 participants (KOA: n=10, 50%; healthy controls: n=10, 50%) completed 3 standardized walking conditions (self-paced, fast-paced, and endurance-equivalent). IMU-derived gait parameters were compared with motion capture at the stride level (≥5000 strides) using Pearson correlation, intraclass correlation coefficients (ICCs), mean absolute error, root mean square error, and Bland-Altman analysis. Participants completed the Stanford Brief Activity Survey to categorize PA level. Secondary analyses emphasized effect sizes and 95% CIs given the pilot design. Feasibility was assessed by protocol completion, data completeness, and tolerability.

**Results:**

IMU-derived gait speed demonstrated strong agreement with motion capture (*r*=0.99; ICC=0.98, 95% CI 0.95‐0.99; mean absolute error=0.07 m/s; bias=0.01 m/s; 95% limits of agreement −0.14 to 0.16 m/s). Stride length and cadence showed similarly high agreement (ICC range 0.96‐0.97). All participants completed the protocol with minimal adverse events. Exploratory analyses suggested that participants reporting higher PA demonstrated directionally faster gait speed and longer stride length, with small-to-moderate effect sizes, whereas inactive participants exhibited higher stance-related rhythm parameters.

**Conclusions:**

Foot-mounted IMUs demonstrated strong stride-level agreement with motion capture across walking conditions, supporting technical validity in both KOA and healthy populations. Exploratory findings suggest that wearable-derived gait parameters may reflect activity-related differences; however, larger longitudinal studies are required to confirm these preliminary signals.

## Introduction

Knee osteoarthritis (KOA) is a prevalent musculoskeletal condition characterized by chronic pain, joint degeneration, and progressive mobility limitations [1-3]. Clinical evaluation commonly relies on radiographic imaging and patient-reported outcome measures (PROMs); however, these tools do not fully capture real-world movement impairments during daily activities [3]. Objective gait assessment may therefore provide complementary information regarding functional limitation and disease impact in individuals with KOA.

Wearable inertial measurement units (IMUs) offer a scalable and portable method for quantifying spatiotemporal gait parameters outside of specialized gait laboratories [4-8]. A growing body of work has demonstrated the analytical validity of IMU-based systems for detecting gait events and reconstructing spatiotemporal parameters across laboratory and real-world environments [5-12]. Validation studies have shown strong agreement between ankle- or foot-mounted IMUs and reference systems, including optical motion capture and electronic walkways, for parameters such as gait speed, stride length, cadence, and event timing [10-18]. In KOA populations specifically, wearable accelerometers and IMUs have been used to quantify reduced overall activity levels, slower walking speeds, altered stride length, and increased gait variability compared with healthy controls [5,7,8,10,19,20].

Several prior studies have provided concrete demonstrations of wearable-based gait assessment in KOA and related populations [21-26]. For example, waist-mounted accelerometers such as the ActiGraph system have been used in free-living conditions to quantify step counts and activity intensity, demonstrating reduced overall activity levels and slower habitual walking speeds among individuals with KOA compared with healthy controls [21-24]. Laboratory-based investigations using foot- or ankle-mounted IMUs, including systems such as Shimmer and G-Walk wireless inertial sensors, have reported shorter stride length, altered cadence, and increased gait variability in KOA cohorts, with several studies validating spatiotemporal parameters against optical motion capture or electronic walkways [16,21-26]. Collectively, these findings support the feasibility and analytical validity of wearable-based gait assessment in KOA.

However, important methodological and conceptual gaps remain. First, much of the prior work has either emphasized device validation in controlled settings or cross-sectional group comparisons between KOA and healthy individuals, rather than integrating multicondition motion capture validation with activity-stratified subgroup analyses within the same study framework.

Second, it remains unclear whether wearable-derived gait parameters meaningfully vary across self-reported physical activity (PA) strata within KOA and healthy populations. Self-report instruments such as the Stanford Brief Activity Survey (SBAS) categorize individuals by perceived activity intensity but may not fully reflect objective gait performance [5-10]. Prior research in older adults and community-dwelling populations suggests that wearable-derived gait metrics can detect differences associated with activity level and mobility capacity [27-31]. However, whether these sensor-based parameters discriminate between sedentary and active individuals within KOA cohorts—particularly when validated against motion capture—has not been well characterized.

To address these gaps, we conducted a pilot cross-sectional validation study with exploratory subgroup analyses. The primary objective was to evaluate the technical validity of foot-mounted IMU–derived gait parameters compared with gold-standard optical motion capture across standardized walking conditions. The secondary exploratory objective was to assess whether IMU-derived gait parameters differed across self-reported PA levels and KOA status. We focused on pace-domain metrics (eg, gait speed and stride length), which reflect movement efficiency and mobility, and rhythm-domain metrics (eg, stance ratio, push ratio, and foot flat ratio), which capture transitional mechanics and gait stability relevant to KOA pathology.

As a pilot study, our goal was not to establish definitive causal relationships but to evaluate feasibility, quantify agreement metrics using multiple complementary methods, and generate preliminary effect size estimates to inform future adequately powered investigations leveraging wearable technology in musculoskeletal populations.

## Methods

### Study Recruitment and Eligibility

We enrolled 10 individuals with clinically documented KOA (Kellgren-Lawrence grades 2‐4) and 10 healthy controls. Individuals aged 18 to 90 years with a confirmed diagnosis of KOA were eligible. Healthy controls were required to have no history of lower extremity musculoskeletal pathology affecting gait. Exclusion criteria included severe cardiopulmonary disease, neurologic, or orthopedic conditions associated with substantial mobility impairment.

### Ethical Considerations

All participants provided written informed consent. The study was approved by the Stanford University institutional review board (IRB #64107) and conducted in accordance with Health Insurance Portability and Accountability Act (HIPAA) guidelines.

### Feasibility Assessment

Feasibility was defined a priori as (1) successful completion of all walking trials, (2) adequate IMU data capture for stride-level analysis, and (3) tolerability of sensor placement without serious adverse events. These criteria were selected to evaluate protocol adherence, sensor tolerability, and adequacy of wearable data capture for stride-level gait analysis.

All participants completed the protocol. Two participants reported mild discomfort or transient skin irritation related to sensor placement. Sufficient stride-level data were collected from all participants for analysis.

### Sample Size Justification

This study was designed as a pilot technical validation study rather than a powered clinical comparison trial. Sample size was guided by prior IMU validation studies, which commonly included 10 to 25 participants when repeated stride-level observations are analyzed using mixed-effects modeling [32-36].

The primary outcome was agreement between IMU-derived gait parameters and motion capture. Because each participant contributed multiple strides across walking conditions, repeated stride-level observations improved precision of agreement estimates while mixed-effects models accounted for within-participant clustering. The study was not powered to detect differences across KOA phenotypes or disease subtypes. Exploratory subgroup analyses were hypothesis-generating and intended to inform effect size estimation for future trials.

### Gait Assessment Protocol

Participants completed three standardized walking conditions: (1) self-paced walking test (SPWT), (2) 40-m fast-paced walking test (40mFPWT), and (3) the 6-minute walk test (6MWT). For the 6MWT, wearable IMU data were recorded continuously for the entire duration of the walk. During exploratory algorithm development, shorter analysis windows within the 6MWT were evaluated to approximate the temporal duration of the SPWT and 40mFPWT trials and to ensure comparability of gait features across tasks. Because gait parameters derived from these windows were consistent with those derived from the full walk, the final analyses used stride-level data from the complete 6MWT recording to maximize the number of observed strides and improve parameter stability. These tests were selected based on prior work demonstrating that endurance, self-paced, and fast-paced walking differentially influence pace- and rhythm-domain gait characteristics. Protocol intake form can be found in Table S1 in Multimedia Appendix 1.

### IMU Data Acquisition

**Figure 1.**
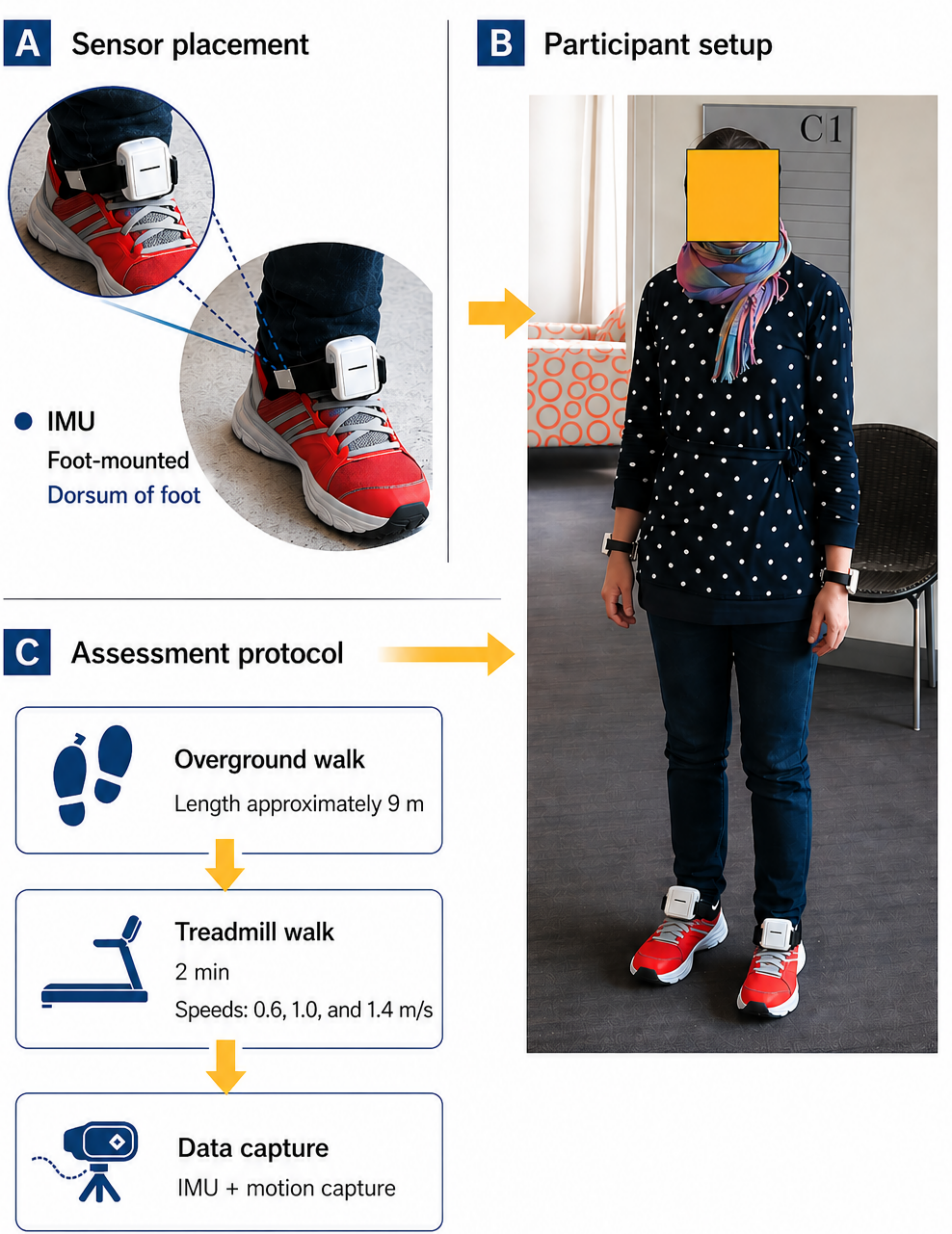
Sensor placement and experimental setup. (A) Inertial measurement unit (IMU) placement on the dorsum of the foot. (B) Participant setup during standardized walking trials. (C) Overview of gait assessment protocol and data capture workflow.

Gait data were collected using the Shimmer3 wearable IMU platform (Shimmer Sensing), which incorporates triaxial accelerometer and gyroscope sensors [28,31,35]. Sensors were mounted bilaterally on the dorsum of each foot using elastic straps to minimize motion artifact and optimize detection of gait events (Figure 1). Data were sampled at 102.4 Hz and hardware synchronized through the control software. Prior to processing, raw signals were resampled to 200 Hz using linear interpolation to align with the validated algorithmic pipeline used for gait parameter extraction [28,31,35,36].

Spatiotemporal gait parameters were derived using previously validated IMU-based algorithms that detect heel strike and toe-off events from foot angular velocity and acceleration signals. These gait events were used to reconstruct stride-level spatiotemporal parameters including gait speed, stride length, cadence, and stance-phase timing. Extracted parameters included gait speed, stride length, cadence, heel strike timing, toe-off timing, peak angular velocity, stance ratio, push ratio, and foot flat ratio. Pace-domain parameters (eg, gait speed, stride length, and peak angular velocity) were selected to reflect movement efficiency and mobility function, whereas rhythm-domain parameters (eg, stance ratio, push ratio, and foot flat ratio) were selected to capture gait stability and transitional mechanics relevant to individuals with KOA.

### Motion Capture Validation

To evaluate technical validity, IMU-derived gait parameters were compared against a laboratory-based optical motion capture reference system (Vicon Motion Systems Ltd) in all 20 participants. Each participant completed 3 trials under each walking condition (self-paced, fast-paced, and endurance), resulting in 9 trials per participant and 180 total trials across the cohort. Conservatively assuming a minimum of 30 strides per trial, approximately 5000 strides were included in validation analyses. Stride-level data were derived from the full duration of each walking trial, including the complete 6MWT rather than truncated analysis windows.

Motion capture data were sampled at 200 Hz. IMU and motion capture systems were time-aligned using a shared trigger signal generated at the start of each trial to ensure precise temporal synchronization. Spatiotemporal parameters were independently derived from IMU-based validated algorithms and from marker-based kinematic reconstruction using motion capture data.

### Statistical Agreement Analysis (Primary Aim)

Agreement between IMU-derived and motion capture–derived parameters was assessed using Pearson correlation coefficients to evaluate linear association and intraclass correlation coefficients (ICC; 2-way mixed-effects model with absolute agreement) to quantify reliability. Absolute error was evaluated using mean absolute error (MAE) and root mean square error (RMSE). Bland-Altman analysis was performed to assess systematic bias and calculate 95% limits of agreement. All validation analyses were conducted at the stride level. The primary analytic unit for validation analyses was the individual stride**,** with repeated strides nested within participants and walking trials. Mixed-effects models accounted for clustering of strides within participants and across walking conditions.

### PA and Patient-Reported Measures

Participants completed the SBAS, the 36-Item Short Form Health Survey (SF-36), the Western Ontario and McMaster Universities Arthritis Index (WOMAC), and the Knee Injury and Osteoarthritis Outcome Score (KOOS) [28,37,38]. The SBAS categorized participants as inactive (0) or active (1) based on self-reported PA level.

### Exploratory Clinical Analyses (Secondary Aim)

Given the pilot sample size, exploratory analyses emphasized effect sizes (Cohen *d*) and 95% CIs rather than a dichotomous interpretation of statistical significance. Between-group comparisons were conducted using ANOVA or nonparametric equivalents when distributional assumptions were not met. Multivariable linear models adjusting for age, sex, BMI, and race were used to assess the directional stability of observed associations. No interaction terms were included, and no data-driven variable selection procedures were performed.

Because the secondary analyses were exploratory and hypothesis-generating, no formal adjustment for multiple comparisons was applied. All analyses were conducted using SAS (version 9.4; SAS Institute). Statistical significance was defined as *P*<.05, although primary interpretation focused on effect sizes and precision estimates.

## Results

### Feasibility Outcomes (Participant Level)

Of 40 individuals screened, 30 (75%) met eligibility criteria, and 20 (50%) were enrolled (n=10, 50% KOA and n=10, 50% healthy controls). All enrolled participants completed the full protocol, including all 3 walking tests (SPWT, 6MWT, and 40mFPWT) and all PROMs.

Missing IMU data were minimal (<3%) and were handled during preprocessing using the validated interpolation pipeline, without loss of analytic variables. No survey data were missing.

Two minor wearable-related adverse events were observed (mild skin irritation and rash), both resolved with modification of the foot strap. There were no falls, cardiopulmonary events, or study withdrawals. Recruitment targets were achieved within the planned time frame, and no participants were lost to follow-up. These findings support the feasibility, safety, and tolerability of foot-mounted IMU-based gait assessment in adults with KOA and healthy controls (Table 1).

**Table 1.** Feasibility assessment*.*

Feasibility metric	Participants, n (%)
Eligible (n=40)	30 (75)
Enrolled (n=40)	20 (50)
Completed study protocol (n=20)	20 (100)
Completion of SPWT^a^ (n=20)	20 (100)
Completion of 6MWT^b^ (n=20)	20 (100)
Completion of 40mFPWT^c^ (n=20)	20 (100)
Completion of all PROMs^d^ (n=20)	20 (100)
Reasons for nonparticipation (n=10)	
Lost device	2 (20)
Declined participation	3 (30)
Scheduling or time burden	3 (30)
Pain or fatigue	2 (20)
Data completeness and safety (n=20)	
Participants with missing IMU^e^ data^f^	0 (0)
Participants with missing survey data	0 (0)
Participants with adverse events	2 (10)^g^
Participants requiring protocol deviations	2 (10)^h^
Follow-up (n=20)	
Completed follow-up	20 (100)^i^
Withdrawals	0 (0)

a SPWT: self-paced walking test.

b 6MWT: 6-minute walk test.

c 40mFPWT: 40-m fast-paced walking test.

d PROM: patient-reported outcome measure.

e IMU: inertial measurement unit.

f Intermittent signal loss (<3%) was corrected during preprocessing without participant-level data loss.

g Skin irritation (n=1) and mild rash (n=1).

h Modified protocol with alternate foot strap for patients with pain and rash.

i Achieved target sample within planned time frame.

### Primary Aim: Motion Capture Validation

IMU-derived parameters were computed from the complete duration of each walking trial, including the full 6MWT, yielding a large stride-level dataset for validation analyses. Agreement metrics remained stable across walking conditions, supporting the validity of the IMU-derived gait algorithm under varying walking speeds and endurance demands.

IMU-derived gait speed demonstrated very strong agreement with the optical motion capture reference (Table 2). Pearson correlation was 0.99, and the ICC (2-way mixed-effects model with absolute agreement) was 0.98 (95% CI 0.95‐0.99). The MAE was 0.07 m/s, and RMSE was 0.09 m/s. Bland-Altman analysis showed minimal systematic bias, with a mean bias of 0.01 m/s and 95% limits of agreement ranging from −0.14 to 0.16 m/s. No evidence of proportional bias was observed across the range of walking speeds (Table 2).

**Figure 2.**
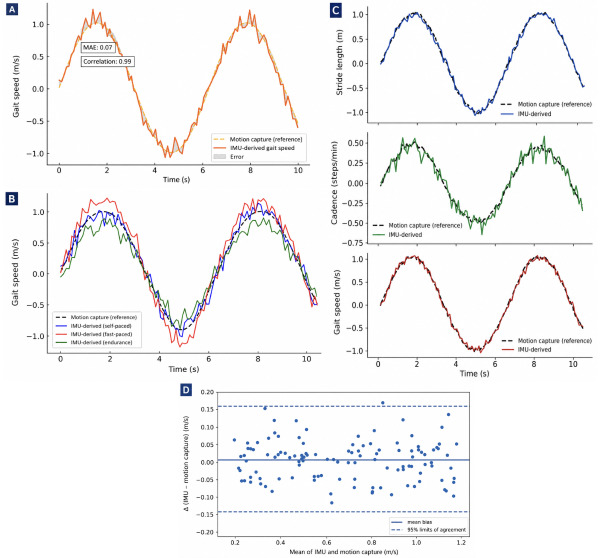
Validation of the inertial measurement unit (IMU)–derived gait algorithm against a gold-standard motion capture system across different walking conditions and gait parameters. (A) Gait speed comparison between IMU and motion capture. (B) Gait speed across walking conditions. (C) Representative stride-level agreement for stride length, cadence, and gait speed. (D) Bland-Altman plot showing agreement and 95% limits of agreement (LOA).

Similarly strong agreement was observed for stride length and cadence. For stride length, Pearson correlation was 0.97 and ICC was 0.96 (95% CI 0.94‐0.98), with an MAE of 0.016 m. For cadence, Pearson correlation was 0.98 and ICC was 0.97 (95% CI 0.93‐0.99), with an MAE of 1.1 steps/min. Bland-Altman analyses for both parameters demonstrated narrow limits of agreement without systematic drift (Table 2; Figure 2).

**Table 2.** Agreement between inertial measurement unit (IMU)–derived and motion capture–derived gait parameters*.*

Parameter	*r*	ICC^a^ (95% CI)	MAE^b^	RMSE^c^	Bias	95% LOA^d^
Gait speed (m/s)	0.99	0.98 (0.95‐0.99)	0.07	0.09	0.01	−0.14 to 0.16
Stride length (m)	0.97	0.96 (0.94‐0.98)	0.016	0.021	0.000	−0.05 to 0.05
Cadence (steps/min)	0.98	0.97 (0.93‐0.99)	1.1	1.5	0.0	−4.0 to 4.0

a ICC: intraclass correlation coefficient.

b MAE: mean absolute error.

c RMSE: root mean square error.

d LOA: limits of agreement.

Heel strike and toe-off timing demonstrated high temporal precision, with mean absolute timing differences of approximately 10 milliseconds between IMU-derived and motion capture–derived events. Agreement remained stable across walking conditions and between KOA and healthy participants. Collectively, these findings support the technical validity of stride-level IMU-derived gait parameters. Figure 2 shows validation of IMU-derived gait parameters with a gold-standard motion capture system and across different walking conditions and sample gait metrics. There was high agreement with the gold standard (correlation=0.99, MAE=0.07 m/s).

### Secondary Aim: Exploratory Differences by Self-Reported PA

**Figure 3.**
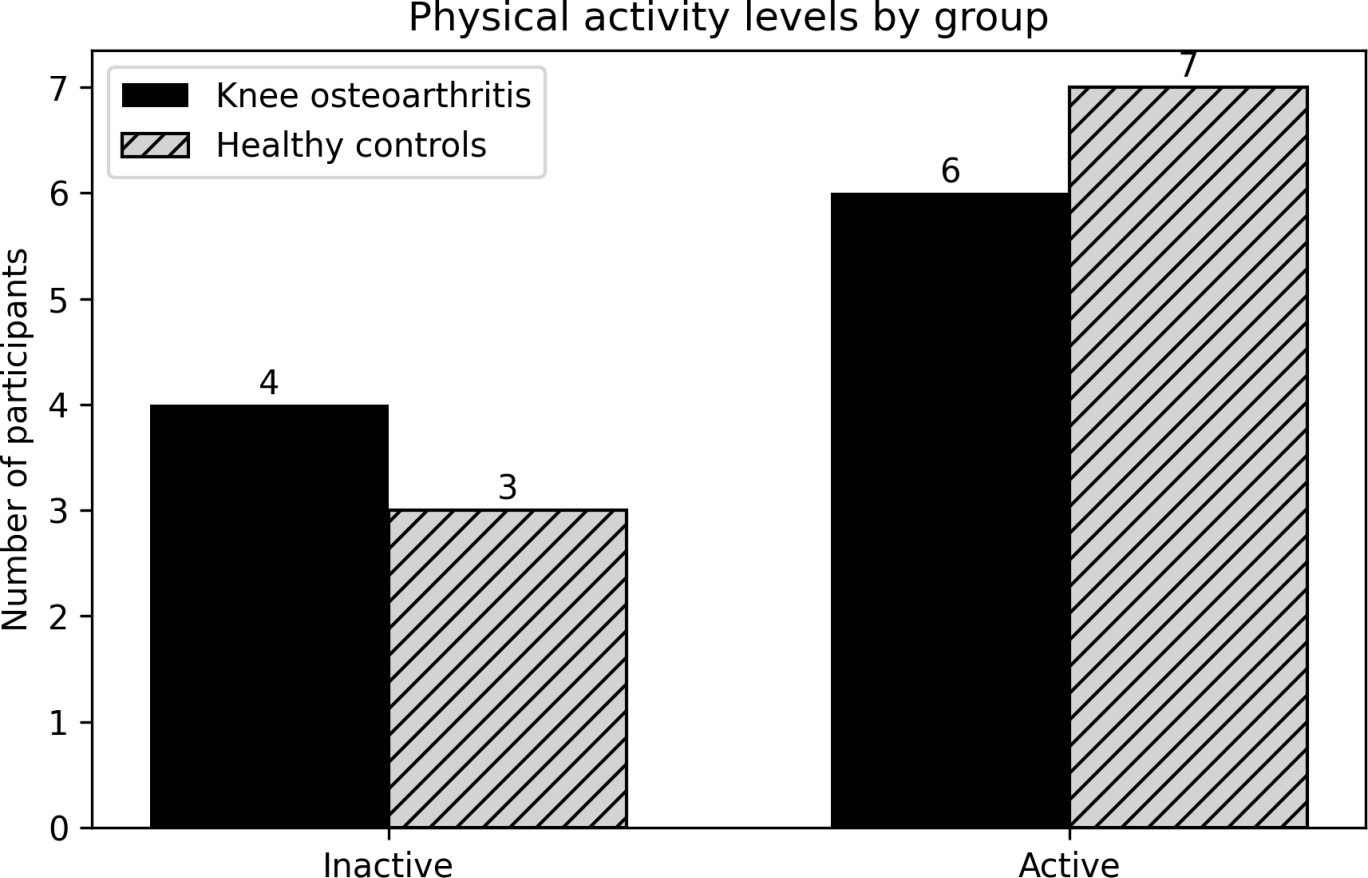
Distribution of activity levels in individuals with knee osteoarthritis vs healthy controls. The bar chart compares the number of participants classified as inactive or active between individuals with knee osteoarthritis and healthy controls. No significant differences in activity distribution were observed between groups.

Exploratory analyses were conducted at the participant level (n=20) and interpreted as hypothesis-generating. Participants were categorized as inactive or active based on the SBAS, and the distribution of activity levels did not differ between KOA and healthy groups (Figure 3).

Across walking conditions, higher self-reported PA was directionally associated with faster gait speed, greater stride length, and higher peak angular velocity (Table 3). Effect sizes for these comparisons ranged from small to moderate (Cohen *d* approximately 0.44‐0.72), although precision was limited given the pilot sample size.

**Table 3.** Effect sizes for inertial measurement unit (IMU)–derived gait features across walking tasks (inactive vs active participants)^a^.

IMU-derived gait feature	6MWT	SPWT	40mFPWT
Push ratio (%)	0.45	0.35	0.18
Foot flat ratio (%)	0.35	0.34	—^b^
Lift-off angle	0.33	0.33	0.32
Gait speed	0.31	0.45	0.25
Stride length	0.34	0.44	0.27
Angular velocity	0.29	0.30	0.26
Foot speed at MTC^c^	0.28	0.42	—
Foot speed at stride length MTC	—	—	0.22
Double support	0.23	0.30	—
Stance	0.19	0.24	0.18
Heel strike angle	0.25	0.23	—
Minimum toe clearance	—	0.32	—
Load ratio (%)	—	0.22	—

a Effect sizes represent standardized mean differences between inactive and active participants derived from wearable IMU gait metrics across 3 walking tasks: the 6-minute walk test (6MWT), self-paced walking test (SPWT), and 40-m fast-paced walking test (40mFPWT). For the 6MWT, gait features were calculated using the entire recorded walk, which provided the largest stride sample for analysis.

b Dashes indicate gait features that were not applicable to the corresponding walking task.

c MTC: minimum toe clearance.

Inactive participants demonstrated directional differences in both pace- and rhythm-domain parameters, including lower gait speed, shorter stride length, higher foot flat ratio, altered push ratio, and increased heel strike angle (Table 3). These findings suggest that wearable-derived gait metrics may capture activity-related biomechanical adaptations. However, given the limited sample size and exploratory design, these estimates should be interpreted cautiously.

### Adjusted Analyses (Directional Stability Assessment)

Prespecified multivariable models adjusting for age, sex, BMI, and race were conducted separately for each walking condition to assess directional stability of the observed associations (Table 4). Across the SPWT, 40mFPWT, and 6MWT, pace-domain parameters remained directionally higher in active participants, whereas rhythm-related parameters such as foot flat ratio and stance ratio tended to remain higher in inactive participants. No interaction terms were included, and models were intentionally parsimonious to minimize overfitting.

Given the pilot nature of the study and the absence of formal multiplicity correction, adjusted findings are interpreted as exploratory and hypothesis-generating rather than confirmatory.

**Table 4.** Adjusted linear regression models evaluating associations between self-reported physical activity (active vs inactive) and gait parameters across walking conditions^a^.

Walking condition and outcome		Active vs inactive), β (95% CI)		Standardized effect (Cohen *d*)	*P* value^b^
SPWT^c^					
Gait speed (m/s)		0.09 (−0.02 to 0.20)		0.58	.10
Stride length (m)		0.05 (−0.01 to 0.11)		0.61	.08
Foot flat ratio		−0.04 (−0.10 to 0.02)		0.47	.15
40mFPWT^d^					
Gait speed (m/s)		0.12 (0.01 to 0.23)		0.72	.04
Peak angular velocity		8.5 (−1.2 to 18.2)		0.63	.09
6MWT^e^					
Gait speed (m/s)		0.07 (−0.03 to 0.17)		0.44	.16
Stance ratio		−0.03 (−0.09 to 0.03)		0.41	.21

a Models adjusted for age, sex, BMI, and race.

b *P*<.05.

c SPWT: self-paced walking test.

d 40mFPWT: 40-m fast-paced walking test.

e 6MWT: 6-minute walk test.

## Discussion

### Principal Findings

This pilot study evaluated the feasibility and technical validity of foot-mounted IMUs for quantifying spatiotemporal gait parameters in adults with KOA and healthy controls. Motion capture validation demonstrated strong agreement between IMU-derived and reference measures across standardized walking conditions. Agreement was evaluated using multiple complementary metrics, including ICCs, absolute error measures, and Bland-Altman analyses. Across ≥5000 strides pooled at the stride level, IMU-derived gait speed, stride length, and cadence showed high concordance with motion capture, minimal systematic bias, and narrow limits of agreement. These findings support the technical validity of the IMU-based gait algorithm across both healthy and pathological gait patterns.

In addition to validation, this study assessed the feasibility of deploying wearable sensors in adults with KOA. Recruitment targets were achieved within the planned time frame, all enrolled participants completed the protocol, and missing data were minimal. These findings suggest that high-frequency IMU-based gait assessment is operationally feasible in this population.

Exploratory analyses examined whether self-reported PA levels were directionally associated with IMU-derived gait parameters. Contrary to the initial hypothesis, self-reported PA distribution did not differ substantially between KOA and healthy participants. However, across walking conditions, individuals reporting higher activity levels demonstrated directionally faster gait speed and longer stride length, whereas inactive participants tended to exhibit differences in rhythm-related parameters such as foot flat ratio and stance-related metrics. Effect sizes were generally small to moderate, but CIs were wide, reflecting the pilot sample size. These associations should therefore be interpreted as preliminary signals rather than definitive group differences.

The observed directional patterns are broadly consistent with prior laboratory-based gait research demonstrating reduced gait speed and altered stance mechanics in individuals with KOA [39-43]. The literature indicates that sedentary individuals, who often have greater joint impairments, show higher heel strike angle, peak ankle angular velocity, push ratio, and foot flat ratio, concordant with prior optical gait lab studies [42-45]. Our exploratory analyses support the literature showing that sedentary individuals spent more time in the stance phase of gait, reflected by higher push and foot flat ratios [42,43]. Other studies using optoelectrical gait analysis have linked KOA to reduced knee range of motion, increased ankle varus moment, and higher knee adduction moment—all factors that increase medial knee joint loading and KOA progression [42,43].

However, because this study relied on foot-mounted sensors, we did not assess joint-level kinematics or loading parameters such as knee adduction moment. Accordingly, mechanistic inferences regarding joint loading or disease progression cannot be made from the present data. Nonetheless, observations from this study could help inform follow-up future investigations. During fast-paced (40mFPWT) and endurance (6MWT) walking, sedentary participants demonstrated directionally greater lift-off angle, higher foot flat ratio, and increased peak ankle angular velocity compared with active participants. Prior literature has reported that increased lift-off angle during the swing phase may represent a compensatory strategy to offload a symptomatic limb in individuals with KOA [42-44]. While we did not directly measure joint loading or knee kinetics, the directional consistency of these sensor-derived parameters with previously described compensatory adaptations suggests that wearable IMUs may be sensitive to activity-related biomechanical differences observed under more demanding walking conditions.

These findings should be interpreted as hypothesis-generating rather than mechanistic, but they provide preliminary support for the ability of foot-mounted sensors to detect gait adaptations that have been described in laboratory-based motion analysis studies. Beyond condition-specific gait differences, sensor-derived metrics may also complement PROMs by capturing aspects of functional performance not fully reflected in self-report measures [2-6,10,11].

Prior research has demonstrated discordance between reported knee pain and objective measures of physical function, muscle strength, and joint range of motion in individuals with KOA [44-46]. In this context, IMU-based assessments may help bridge the gap between perceived and observed function by providing objective, stride-level data on movement efficiency and gait mechanics. Larger longitudinal studies will be needed to determine how these objective gait markers relate to symptom burden, physical confidence, and clinical outcomes over time.

### Study Limitations

This study has several limitations. First, the participant-level sample size was small (n=20), and although stride-level validation analyses were robust, exploratory group comparisons are subject to imprecision. Second, multiple related gait parameters were examined across several walking conditions. Although emphasis was placed on effect sizes and uncertainty rather than statistical significance, formal adjustment for multiplicity was not performed given the pilot design. Third, the cross-sectional design precludes causal inference regarding the relationship between PA and gait characteristics.

Despite these limitations, our findings demonstrate that wearable IMUs can generate technically valid and high-resolution gait data in adults with KOA. These pilot data inform effect size estimation and analytic strategies for future adequately powered longitudinal studies designed to evaluate clinical relevance, responsiveness to intervention, and relationships between objective gait metrics and patient-reported outcomes.

### Conclusions

In this pilot cross-sectional validation study, foot-mounted wearable IMUs demonstrated strong agreement with optical motion capture across standardized walking conditions, with high ICCs and minimal systematic bias. These findings support the technical validity and feasibility of stride-level gait assessment using foot-mounted wearable IMUs in adults with KOA.

Exploratory analyses suggested directional differences in pace- and rhythm-domain gait parameters across self-reported PA levels. However, given the small sample size, cross-sectional design, and absence of multiplicity adjustment, these associations should be interpreted as hypothesis-generating.

Larger, longitudinal investigations are needed to confirm these preliminary signals and to determine how wearable-derived gait parameters relate to disease severity, PA behavior, and clinically meaningful outcomes over time.

## Supplementary material

10.2196/80728 Multimedia Appendix 1 Structured data collection form.

## Abbreviations

40mFPWT: 40-m fast-paced walking test
6MWT: 6-minute walking test
HIPAA: Health Insurance Portability and Accountability Act
ICC: intraclass correlation coefficient
IMU: inertial measurement unit
KOA: knee osteoarthritis
KOOS: Knee Injury and Osteoarthritis Outcome Score
MAE: mean absolute error
PA: physical activity
PROM: patient-reported outcome measure
RMSE: root mean squared error
SBAS: Stanford Brief Activity Survey
SF-36: 36-Item Short Form Health Survey
SPWT: self-paced walking test
WOMAC: Western Ontario and McMaster Universities Arthritis Index
