# Supplementary material for: Wearable Sensor–Derived Gait Parameters Across Self-Reported Physical Activity Levels in Individuals With Knee Osteoarthritis and Healthy Controls: Pilot Cross-Sectional Validation Study
Source: JMIR Form Res. 2026 Jun 3;10:e80728. doi: 10.2196/80728 (PMC13233006; doi:10.2196/80728)
Supplement: Multimedia Appendix 1 [file formative-v10-e80728-s001.docx]

Supplementary Tables

***Supplementary Table 1.*** Structured Data Collection Form Used During IMU-Based Gait Testing

| **Section** | **Item** | **Description / Fields Captured** |
| --- | --- | --- |
| **Participant Information** | Study ID | Unique participant identifier |
|  | KOA Status | KOA (KL grade 2–4) / Healthy Control |
|  | Age (years) | Continuous variable |
|  | Sex | Male / Female |
|  | BMI (kg/m²) | Calculated from height and weight |
|  | Race/Ethnicity | Self-reported |
| **Sensor Setup** | Sensor Type | Shimmer3 IMU (triaxial accelerometer + gyroscope) |
|  | Sampling Rate | 102.4 Hz |
|  | Placement Location | Bilateral dorsum of foot |
|  | Strap Tolerance | Tolerated / Modified strap used |
|  | Signal Check | Verified synchronization and data capture prior to trial |
| **Walking Conditions** | Self-Paced Walking Test (SPWT) | Completed (Yes/No); Trial duration recorded |
|  | 40m Fast-Paced Walk (40mFPWT) | Completed (Yes/No); Trial duration recorded |
|  | 6-Minute Walk Test (6MWT) | Completed (Yes/No); Distance recorded |
| **Gait Event Capture** | Heel Strike Detection | Confirmed (Yes/No) |
|  | Toe-Off Detection | Confirmed (Yes/No) |
| **Protocol Adherence** | Trial Completion | All trials completed (Yes/No) |
|  | Deviations | Description if applicable |
| **Adverse Events** | Skin Irritation | None / Mild / Moderate |
|  | Rash | Yes/No |
|  | Fall / Near Fall | Yes/No |
|  | Cardiopulmonary Event | Yes/No |
| **Data Integrity** | IMU Signal Dropout | % Missing (<3%) |
|  | Interpolation Applied | Yes (validated pipeline) |
|  | Survey Completion | WOMAC / KOOS / SF-36 / SBAS completed (Yes/No) |

**Multimedia Appendix 1.** Standardized gait assessment protocol form used during IMU-based walking assessments. The structured form was completed at each study visit to document participant characteristics, sensor setup verification, walking test completion, protocol adherence, adverse events, and data integrity checks.

This structured documentation was completed prospectively at the time of testing to ensure protocol fidelity and reproducibility.

***Supplementary Table 2.*** Relationships between knee OA-specific measures and subscales of the SF-36*.*

|  | **KOOS-Pain** | **KOOS-Symptom** | **KOOS-ADL** | **KOOS-Sports/Rec** | **KOOS-QOL** | **Pain‡** | **Joint Stiffness‡** | **Physical Function‡** | **WOMAC** |
| --- | --- | --- | --- | --- | --- | --- | --- | --- | --- |
| SF-36 Subscales |  |  |  |  |  |  |  |  |  |
| Physical Functioning | 0.59 | 0.11 | 0.31 | 0.33 | 0.36 | -0.36 | 0.01 | -0.32 | -0.29 |
| Vitality | 0.31 | -0.36 | -0.22 | 0.27 | 0.05 | 0.08 | 0.18 | 0.32 | 0.24 |
| Mental Health | 0.21 | -0.26 | -0.10 | 0.31 | -0.02 | -0.10 | 0.03 | 0.13 | 0.02 |
| Social Functioning | 0.78** | 0.08 | -0.07 | 0.52 | 0.67* | -0.22 | -0.17 | 0.22 | 0.04 |
| Bodily Pain | 0.66* | 0.03 | -0.08 | 0.17 | 0.28 | -0.29 | -0.15 | -0.09 | -0.14 |
| General Health | 0.59 | -0.07 | -0.30 | 0.15 | 0.10 | -0.14 | -0.11 | 0.12 | 0.04 |

p-values for the correlations: **p <.01 and *p<0.05; ‡reflects subscales of the WOMAC index.

***Supplementary Table 3.* Bonferroni corrections for multiple comparisons**

| **Comparison Type** | **Number of Tests** | **Unadjusted Alpha** | **Bonferroni-Adjusted Alpha** | **Correction Applied** |
| --- | --- | --- | --- | --- |
| IMU-Gait Parameters (Rhythm) | 50 | 0.05 | 0.001 | Bonferroni |
| IMU-Gait Parameters (Pace) | 50 | 0.05 | 0.001 | Bonferroni |
| Self-Reported Activity (SBAS) | 20 | 0.05 | 0.0025 | Bonferroni |
| Spearman Correlations | 30 | 0.05 | 0.0017 | Bonferroni |
| Between-Group Comparisons (KOA vs. Healthy) | 25 | 0.05 | 0.002 | Bonferroni |
| Gait Test Conditions (SPWT, 6MWT, 40mFPWT) | 10 | 0.05 | 0.005 | Bonferroni |
|  |  |  |  |  |

Supplementary Figures

**Supplementary Figure 1. Distribution of Wilcoxon Rank Sum Test Scores for Physical Function Subscale of SF3-6**

X-axis shows healthy controls vs. knee OA group and Y-axis shows Wilcoxon rank sum scores.

Differences between healthy and disease populations were evaluated by the Wilcoxon rank sum test, or non-parametric equivalent for one way ANOVA (Kruskal-Wallis Test), P<0.0002.

Figure S1. Physical function differences between KOA and healthy controls.


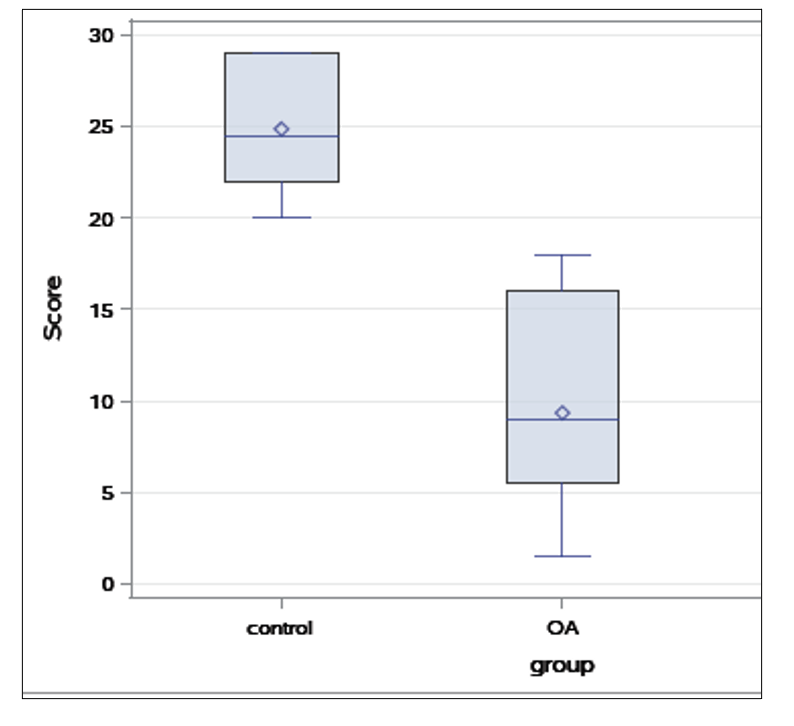


Appendix A. Pace and Rhythm Domains of IMU Gait Parameters Analyzed for Each Foot in One Gait Cycle

| **Parameter** | **Unit** | **Description** |
| --- | --- | --- |
| ***Rhythm Domain*** | | |
| Cadence | steps/min | number of steps per minutes |
| Double Support | % | Percentage of the cycle where both feet are on the ground. |
| Stance Ratio | % | Percentage of the cycle during which part of the foot touches the ground |
| Swing Ratio | % | Percentage of the cycle during which the foot is in the air and does not touch the ground |
| Push Ratio | % | Percentage of the stance between foot flat and toe off |
| Foot Flat Ratio | % | Percentage of the stance where the foot is fully flat on the ground. |
| Loading Ratio | % | Percentage of the stance between heel strike and foot flat |
| ***Pace Domain*** | | |
| Stride Length | meter | Distance between two successive heel strikes. |
| Gait Speed | m/sec | Forward strike speed of one cycle. |
| Peak Angular Velocity | deg/sec | Maximum angular velocity during swing phase. |
| Foot Speed at Minimum Toe Clearance | m/sec | Foot speed at minimum clearance from the ground at toe off |
| Heel Strike Angle | degree | Foot angle at heel strike in the sagittal plane. |
| Lift Off Angle | degree | Foot angle at toe off in the sagittal plane. |
| Swing Width | meter | Maximum lateral displacement during swing. |
| Turning Angle | degree | Angle between two successive foot-flat |
